# Salt Tolerance in Sugar Beet: From Impact Analysis to Adaptive Mechanisms and Future Research

**DOI:** 10.3390/plants13213018

**Published:** 2024-10-28

**Authors:** Yuetong Wang, Huajun Liu, Maoqian Wang, Jiahui Liu, Gui Geng, Yuguang Wang

**Affiliations:** 1 Engineering Research Center of Agricultural Microbiology Technology, Ministry of Education, Heilongjiang Provincial Key Laboratory of Ecological Restoration and Resource Utilization for Cold Region, School of Life Sciences, Heilongjiang University, Harbin 150080, China; 2 Heilongjiang Provincial Key Laboratory of Plant Genetic Engineering and Biological Fermentation Engineering for Cold Region, Key Laboratory of Microbiology, College of Heilongjiang Province, Heilongjiang University, Harbin 150080, China; 3 Cash Crops Research Institute of Xinjiang Academy of Agricultural Science (XAAS), Urumqi 830001, Xinjiang, China; 4 National Sugar Crop Improvement Centre, College of Advanced Agriculture and Ecological Environment, Heilongjiang University, Harbin 150080, China

**Keywords:** sugar beet, salt stress, plant physiology, omics technologies, proteomics, transcriptomics, metabolomics

## Abstract

The continuous global escalation of soil salinization areas presents severe challenges to the stability and growth of agricultural development across the world. In-depth research on sugar beet (*Beta vulgaris* L.), an important economic and sugar crop with salt tolerance characteristics, is crucial for to determine its salt-tolerance mechanisms, which has important practical implications for production. This review summarizes the multifaceted effects of salt stress on sugar beet, ranging from individual plant responses to cellular and molecular adaptations. Sugar beet exhibits robust salt-tolerance mechanisms, including osmotic regulation, ion balance management, and the compartmentalization of toxic ions. Omics technologies, including genomics, transcriptomics, proteomics, post-translational modification omics and metabolomics, have played crucial roles in elucidating these mechanisms. Key genes and pathways involved in salt tolerance in sugar beet have been identified, paving the way for targeted breeding strategies and biotechnological advancements. Understanding these mechanisms not only enhances our knowledge of sugar beet’s adaptation strategies but also provides insights for improving salt tolerance in other crops. Future studies should focus on analyzing gene expression changes in sugar beet under salt stress to gain insight into the molecular aspects of its salt-tolerance mechanisms. Meanwhile, the effects of different environmental conditions on sugar beet adaptation strategies should also be investigated to improve their growth potential in salinized soils.

## 1. Introduction

Soil salinity, a multifaceted issue arising from natural and anthropogenic factors, has led to the degradation of over 20% of the world’s farmland [1]. The saline soils in China, covering over 50 million hectares, are divided into two primary regions, namely the inland arid zones and the coastal areas [2]. They predominantly consist of sulfatic- and chloridic-type soils. Sweet beets exhibit a remarkable adaptability to moderately saline sulfatic soils, which are mainly located in the arid inland regions of Xinjiang, Inner Mongolia, and Qinghai [3]. Salted–alkali soils in these areas are characterized by high concentrations of chlorides and sulfates of sodium, potassium, calcium, and magnesium [4,5]. Primary salt is the result of natural evaporation and concentration processes over geological time, forming salt deposits. Secondary salt refers to the soil salinization process caused by unreasonable tillage and irrigation [6,7]. Climate change accelerates surface water evaporation, increasing soil salt concentrations [8]. Poor irrigation practices and river diversion further bring salts to the soil surface [9]. Additionally, excessive chemical fertilizer use and declining water quality exacerbate soil salinization [10]. Soil salinization is a significant issue that affects agricultural productivity. The increasing severity of salinization leads to a decline in soil fertility, disrupting the water absorption process in crops, inhibiting photosynthesis, and hindering the accumulation of nutrients [11]. As a result, the growth capacity of crops is impaired, ultimately leading to significant impacts on agricultural production [12,13,14]. Therefore, a thorough investigation into the mechanisms of salt tolerance in plants can not only unveil the secrets of life adapting to salt stress, but also has profound implications for breeding high-yield, salt-tolerant varieties, restoring the productivity of degraded lands, and establishing sustainable agricultural ecosystems.

Sugar beet (*Beta vulgaris* L.), the second largest sugar crop globally, is a versatile industrial crop [15,16,17,18]. Its roots and leaves provide essential nutrients for animals [19], and yield valuable by-products like molasses and sugar beet meal, which can be converted into biofuels [20,21,22]. Sugar beet contains compounds potentially beneficial for health, aiding in disease prevention [23]. Cultivated from sea beet (*Beta maritima* L.), sugar beet inherits traits such as drought and salt resistance [24,25]. Efforts to understand sugar beet’s salt-tolerance mechanisms and develop tolerant varieties have progressed, although the integration of findings from morphological, physiological, biochemical, and molecular studies remains a challenge [26]. Sugar beet farming plays a pivotal role in sustainable agriculture. Opting for biochar or cattle manure instead of conventional fertilizers during sugar beet cultivation not only enhances sugar yields significantly but also effectively reduces greenhouse gas emissions, demonstrating sugar beet’s positive contribution to advancing sustainable agriculture. By implementing biochar application, we ensure the efficient output of sugar crops while simultaneously safeguarding the ecological environment, achieving a green transformation in agricultural production [27]. Moreover, as part of a crop rotation system, sugar beet synergizes with conservation management practices, contributing to enhanced soil quality, reduced chemical inputs, and the increased stability and productivity of agroecosystems [28]. These characteristics make sugar beet a significant crop choice in tackling challenges such as climate change and soil degradation.

In the post-genomic era, omics technologies—genomics, transcriptomics, proteomics, post-translational modification omics, and metabolomics—are crucial for elucidating plant responses to salt stress [29,30,31]. In recent years, the application of these omics tools in studying plant salt stress has significantly increased, providing valuable insights into the mechanisms of plant responses and adaptations to salinity [32]. In 2013, the staff of the University of Bielefeld, the Spanish Center for Genome Control, and the Max Planck Institute for Molecular Genetics published a high quality article on sugar beet omics. It remarks that sugar beet breeding has entered the omics era [24]. The review evaluates the impact of salt stress on sugar beet, exploring its physiological and molecular responses, and emphasizes the importance of omics tools in understanding salt-stress mechanisms. Omics analyses identify genes associated with salt tolerance and reveal gene expression patterns under stress, crucial for developing salt-tolerant sugar beet varieties and ensuring agricultural productivity.

In the context of the escalating global issue of soil salinization, the originality of this paper manifests in several aspects: Firstly, by systematically summarizing the physiological, cellular, and molecular response mechanisms of sugar beet under salt stress and integrating findings from diverse omics techniques, it furnishes a holistic perspective on its salt tolerance. Secondly, the paper underscores the identification of pivotal genes and metabolic pathways, which lays the groundwork for future precision breeding tactics and biological technology advancements. Additionally, the paper delves into the unique mechanisms of sugar beet’s adaptation to salt stress, such as ion balance regulation and osmolyte synthesis, offering novel directions for research. These innovative points not only consolidate the theoretical foundation of plant salt-tolerance studies but also provide crucial references for improving salt resistance in other crops.

## 2. Effect of Salinity on Sugar Beet

**Figure 1:**
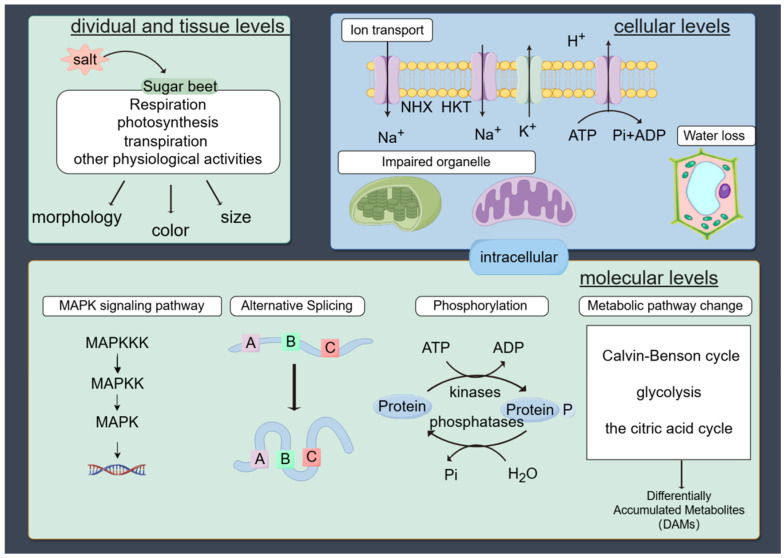
Effect of salt stress on sugar beet from individual growth and development to the molecular level (The figure was drawn using Figdraw: www.figdraw.com, accessed on 18 October 2024).

Sugar beet is notably tolerant to salt compared to other crops, which allows it to efficiently absorb water and nutrients from saline soils [33]. Interestingly, low salt concentrations can enhance the germination and growth rates of sugar beet [34,35]. This enhancement includes promoting root elongation and increased surface area [36] and improved physiological salt resistance indicators [37]. Previous studies found that sugar beet plants withstood a final concentration of 300 mM NaCl in hydroponics for up to 14 days [38], and sugar beet can tolerate up to 500 mM NaCl for seven days without losing viability [39]. This resilience allows sugar beet to maintain productivity on marginal lands like saline–alkali soils, expanding agricultural options. However, exceeding sugar beet salt-tolerance threshold results in salt damage. The damage inflicted by salinity stress on beet manifests across multiple levels, from the individual plant to cellular and molecular processes (Figure 1).

### 2.1. Effect of Salt Stress on Individual and Tissue Levels of Sugar Beet

Salt stress has a comprehensive and profound impact on the individual level of sugar beet [40]. Salt stress disrupts the entire life cycle of the plant, starting from the germination of seeds and extending through to the growth and development of the plant [41]. Germination showed high sensitivity to salt, and studies have shown that salt stress can suppress seed germination to varying degrees [42,43,44]. Even when sugar beet is able to grow under certain levels of salt stress, its growth and development are severely impaired. This is characterized by growth retardation and a reduction in overall volume and biomass [45]. As the main organ of plants undergoing photosynthesis, the leaves also undergo obvious changes under salt stress. There may be a reduction in the number of leaves [19,46]. Additionally, there are morphological changes such as a decrease in leaf area [47,48]. The leaves may also curl, become deformed, and change color to yellow or white [49]. These are the intuitive responses of sugar beet to salt stress. The root system of sugar beet is also adversely affected by high concentrations of salt stress. This can inhibit root elongation and branching, leading to root dysplasia and shorter growth [42]. The overall root distribution is also altered. Collectively, these effects can lead to a decrease in sugar beet’s biomass. It is important to recognize that the impact of salt stress on sugar beet can vary depending on factors such as the extent and timing of the stress. These factors can influence the severity of the effects observed at both the individual and tissue levels of the plant.

### 2.2. Effect of Salt Stress on the Cellular Levels of Sugar Beet

At the cellular level, salt stress affects sugar beet, like other plants, with the following three key stress responses: osmotic stress, oxidative stress, and toxic stress [50]. Salt stress initiates osmotic stress, where elevated external osmotic potential due to high salt concentrations prompts cellular water to move outwards in pursuit of osmotic equilibrium [51]. This leads to cell dehydration and contraction, and the separation of cell membranes and cell walls, affecting cell metabolism and normal physiological functions, and plant wilting [52]. Sugar beet plants have developed the ability to control stomatal closure through guard cells, reducing water evaporation to buffer salt stress [53].

Meanwhile, salt stress will increase the production of reactive oxygen species (ROS) in plants to induce oxidative stress [54]. Excessive ROS can lead to damage to protein, DNA, and other cellular components [55]. Beets, like other plants, have evolved antioxidant systems as defense mechanisms. These systems consist of enzymatic and non-enzymatic components designed to regulate the synthesis and clearance of ROS. The main antioxidant enzymes include superoxide dismutase (SOD), ascorbate peroxidase (APX), catalase (CAT), glutathione peroxidase (GPX), and thioredoxin (Trx) [56,57]. These enzyme rises work together to help sugar beet maintain a balance of ROS levels and prevent excessive damage to targets such as protein, DNA and other cellular components.

Moreover, the toxic stress should not be ignored. Prior research has shown that in saline environments, high concentrations of Na+ and Cl^−^ hinder the absorption of essential ions such as K^+^, Ca^2+^, and Mn^2+^, impacting osmoregulation and the maintenance of cellular turgor [50,58,59,60]. Studies in Arabidopsis have confirmed the inhibitory effect of salt stress on ion uptake and plant growth [61]. Salt stress also damages the cell membrane, altering its permeability and affecting the exchange of substances between the cell and its environment. Prolonged exposure to salt stress can impair the functions of organelles such as mitochondria and chloroplasts [62,63], with impaired mitochondrial function affecting cellular respiration, and chloroplast damage impacting photosynthesis [64], potentially leading to cell death. The severity and duration of these effects determine the extent of salt-stress impact on cells. To counteract these adverse effects, sugar beet cells produce osmoregulatory substances such as proline [65] and increase the energy consumption required by ion pumps to maintain intracellular ion balance [66].

In conclusion, salt stress profoundly impacts the physiological functions of sugar beet cells through osmotic, oxidative, and toxic stresses ultimately possibly leading to cell death.

### 2.3. Effects of Salt Stress on the Molecular Levels of Sugar Beet

At the molecular level, the effects of salt stress on sugar beet are multifaceted. First, it regulates the gene expression patterns [16,67,68]. An example of this is the up- or down regulation of specific genes such as *BvbZIP*, which affects light and ABA signaling pathways [69]. Second, salt stress affects protein synthesis, and at the molecular level, it induces a range of responses in sugar beet plants and affects a variety of vital biological processes. Initially, it regulates changes in gene expression patterns, in which the transcription factors play a central role and directly influence the plant perception and response to salt stress. For example, the downregulation of the *BvbZIP* gene not only changes the dynamic balance of photosynthesis and ABA signaling pathways, but also may affect the synthesis and signaling of plant hormones. Furthermore, the impact of salt stress on protein synthesis within sugar beet is significant and cannot be overlooked. Salt stress exerts a profound influence on the levels of key proteins, thereby regulating various cellular processes in sugar beet. Among these proteins, the 14-3-3 protein family stands out. As a highly conserved protein family in eukaryotic cells, 14-3-3 proteins play a crucial role in various cellular processes by modulating the activity, localization, and stability of other proteins [70,71]. Under salt stress conditions, the levels of 14-3-3 proteins in sugar beet undergo changes, significantly impacting cellular signaling and stress response mechanisms [72], followed by protein phosphorylation. Salt stress can trigger the process of protein phosphorylation, an important way in which protein modification can affect protein function and interactions. For instance, studies have found proteomic differences between salt-sensitive and salt-tolerant beet varieties under salt stress. The results have been shown to involve protein modifications and TCA recurrence. Several proteins for cell-wall synthesis and ROS clearance differ significantly between sensitive and tolerant cultivars, suggesting that these pathways may be highly associated with the salt tolerance of sugar beet [68]. Variable splicing (AS), also known as alternative splicing, is a common biological event in eukaryotes, referring to the process of generating different mRNA isoforms in an mRNA precursor, and an increased diversity of proteins. Although, it is unclear how sugar beet develops AS under salt stress. However, some studies found that among 136 differentially expressed genes identified under alkali stress, unannotated genes were found, of which 24 showed alternative splicing [73].

In addition to protein synthesis, salt stress also disrupts the metabolic process of sugar beet. By regulating the Calvin cycle, glycolysis, and the citric acid cycle, salt stress induces alterations in key metabolic pathways [74]. This disruption leads to changes in the levels of key metabolites, such as reduced sugars, and increases in the levels of compounds such as uridine and serine. These metabolic changes further highlight the complex network of molecular mechanisms that underpin sugar beet’s adaptation to salt stress, emphasizing the intricate and coordinated response of sugar beet plants to environmental challenges. These findings underscore the multifaceted nature of the molecular mechanisms involved in sugar beet’s response to salt stress. They reveal the intricate interplay between gene expression, protein regulation, and metabolic adjustments, which collectively enable sugar beet to adapt and survive under saline conditions.

### 2.4. Effects of Salt Stress on Photosynthesis in Sugar Beet

Salinity stress poses a significant challenge to sugar beet’s photosynthesis, manifested as stomatal closure which aims to reduce water loss; however, this concurrently restricts CO_2_ entry, decelerating photosynthetic rates [46]. High saline conditions induce ion imbalance within plants, particularly insufficient potassium uptake and sodium accumulation, which can be toxic to plant cells. This ion imbalance interferes with cellular functions, including enzyme activity and photopigment efficiency in photosynthesis [50,58,59,60]. Photosynthesis is the process by which plants convert light energy into chemical energy, involving photopigments. Salt stress may cause photopigments to become inactivated or damaged, obstructing light absorption and transfer, thus impairing photosynthetic efficiency [75]. Additionally, the accumulation of salts can lead to chloroplast structural damage, directly affecting chlorophyll levels and the progression of photosynthesis [74]. Salt stress adversely affects photosynthesis from multiple perspectives, and These adverse effects combined decreases in the leaf area, growth, and yield of sugar beet.

## 3. Salt Tolerance and Adaptation Mechanism of Sugar Beet

### 3.1. Basal Adaptation Mechanism of Sugar Beet to Salt Stress

Sugar beet, like other plants, undergoes adaptive morphological changes such as reducing root area and increasing leaf thickness under salt stress [56,76,77,78,79] Simultaneously, sugar beet leaves regulate stomatal opening and closing to minimize water loss through transpiration. At the physiological level, sugar beet maintains cellular osmotic pressure to prevent dehydration by synthesizing and accumulating osmoregulatory substances such as proline, soluble sugars, and organic acids. Furthermore, it enhances its antioxidant defense system to scavenge reactive oxygen species and mitigate oxidative stress-induced cell damage [80,81]. These integrated adaptations enable sugar beet to thrive in challenging environments like saline fields.

### 3.2. Unique Adaptation Mechanism of Sugar Beet to Salt Stress

**Figure 2:**
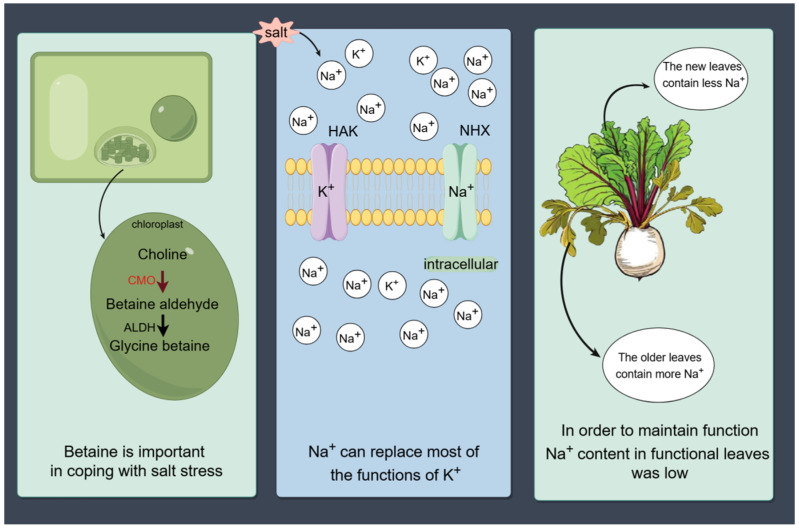
Overview diagram showing the specialized mechanisms of sugar beet in response to salt stress (The figure was drawn by Figdraw: www.figdraw.com, accessed on 18 October 2024).

The unique salt-tolerance mechanism of sugar beet can be categorized into three main strategies (Figure 2). Initially, upon encountering salt stress, sugar beet can rapidly synthesize and accumulate betaine. This accumulation serves a dual purpose, as it effectively regulates the intracellular water potential, alleviating the osmotic stress induced by salt stress, and it mitigates the impact of salt stress on the activity of enzymes involved in photosynthesis [82]. Under salt stress, there is a significant upregulation of genes, proteins, and enzyme activities associated with choline monooxygenase (CMO), which is the enzyme that catalyzes the first step in the synthesis of betaine [83]. These levels return to normal after the plant undergoes rehydration, indicating a direct role for betaine in the molecular response of sugar beet to salt stress for the first time. This response is a critical component of sugar beet’s overall salt-tolerance strategy. By upregulating the production of betaine, sugar beet not only manages the immediate osmotic challenges posed by high salinity but also protects the enzymes crucial for its photosynthetic processes, ensuring its survival and productivity in saline conditions.

Moreover, the plant ion balance of Na^+^, Cl^−^, K^+^ and NO^3−^ is damaged, leading to ion toxicity under salt stress. However, the sugar beet can use Na^+^ to partially replace the K^+^ function. The absorption of Na^+^ can be significantly promoted in a low K^+^ environment, and 95% of K^+^ in sugar beet can grow normally after being replaced by Na^+^ without affecting the yield [84,85]. The current results show that part of the functions in K^+^ can be replaced by Na^+^, such as osmoregulation, auxiliary anion long-distance transport, the control of the stomatal opening, the regulation of enzyme activity, and chloroplast proliferation [86,87].

Finally, sugar beet can respond to salt stress through regionalization mechanisms, locking salt in specific tissues [80]. Under salt stress, sugar beet transports the salt ions absorbed from the environment to the petiole and old leaf storage; when the salt content of functional leaves is low, it has been reported that the content of Na^+^ and Cl^−^ leaf petiole tissue is significantly higher than that of roots or leaves, while Cl^−^ is mainly concentrated in the petiole at a low salt concentration, and is mainly concentrated in the leaves at a high salt concentration [49,88]. This strategy helps to protect functional leaves and enzyme activities to minimize plant damage from salt ions.

## 4. Omics Study of Salt Tolerance in Sugar Beet

Omics analysis has become an important method in the field of plant molecular biology and is widely used to study the molecular mechanisms of stress resistance in plants. Currently, a wide range of omics approaches have been extensively applied to the study of salt-tolerance mechanisms and the functional analysis of salt-tolerance genes in sugar beet.

### 4.1. Transcriptomics of Salt Tolerance in Sugar Beet

Through high-throughput sequencing technology, the researchers conducted a deeper analysis of the gene expression patterns of sugar beet under salt-stress conditions, and revealed a series of genes closely related to the salt-stress response and their regulation rules. Previous studies identified differential gene expression associated with salt tolerance and further identified several key genes in sugar beet in response to salt stress [89,90,91]. These include ion transport [80], osmoregulation [81], antioxidant defense [92], and signal transduction [93]. Sugar beet maintains an intracellular ion balance by upregulating the expression of ion transport genes (*BvNHX1*) to enhance the osmotic ability to resist external high osmotic pressure environment under salt stress [94]. In addition, sugar beet also actively responds to oxidative stress by removing excessive reactive oxygen species by upregulating the expression of the catalase gene, enhancing the activity of the antioxidant enzyme system, and reducing oxidative damage [16]. The identification of the gene *BvM14-SAMS2* as a key enzyme in the biosynthesis of the polyamine precursor S-adenosylmethionine (SAM) underscores its potential role in enhancing salt tolerance in sugar beet [95]. Under salt stress, genes encoding ion pump and channel proteins are also significantly upregulated, underscoring their importance in maintaining intracellular ion balance [67]. A transcriptomic study revealed significant activation of multiple salt-stress-responsive pathways, including starch and sucrose metabolism, alpha-linolenic acid metabolism, phenylpropanoid biosynthesis, and plant hormone signal transduction. These findings indicate that these pathways may play crucial roles in sugar beet’s salt tolerance [96]. Additionally, the aforementioned studies have revealed that the roots and leaves of sugar beet exhibit different adaptive mechanisms in response to salt stress. There are also other studies that can justify this conclusion [97]. Taking CAT as an example, the upregulation of the catalase gene expression in leaves, and, unlike the leaf blade, the downregulated expression of CAT encoding genes in root systems after salt stress can be observed [98]. In conclusion, the transcriptomic studies of salt tolerance in sugar beet provide us with a deep understanding of the mechanisms of sugar beet response to salt stress.

### 4.2. Proteomics of Salt Tolerance in Sugar Beet

Proteomics, a comprehensive investigation into the protein make up and dynamics within living organisms, has greatly advanced our understanding of salt-tolerance mechanisms in sugar beet [99]. In recent years, numerous proteomic studies have shed light on how sugar beet responds to salt stress, revealing intricate molecular networks underlying its adaptability. A proteomic analysis revealed sugar beet’s complex response to salt stress by regulating key proteins, including components of the antioxidant defense system, ion transporters, photosynthetic machinery, and signal transduction proteins [100]. Furthermore, proteomics has identified changes in redox-sensitive proteins in beetroot roots under salt stress. These redox-sensitive proteins are crucial for metabolic regulation, ROS homeostasis, and various cellular functions, such as biosynthesis, signaling, transcription, and photosynthesis [101]. For instance, ferredoxin plays a key role in electron transfer during photosynthesis and helps reduce NADP^+^ to NADPH, thereby managing oxidative stress and supporting biosynthesis [101,102]. Meanwhile, 6-phosphofructokinase 5 (PFK5), a key enzyme in glycolysis, exhibits altered activity under salt stress, highlighting its role in energy metabolism [103]. Conversely, malate dehydrogenase (MDH) levels decrease under salt stress, indicating that salt stress may disrupt the TCA cycle and affect overall energy production [103]. Additionally, thioredoxins (such as Trx Clot and TrxH1) play a significant role in antioxidant defense by reducing disulfide bonds in target proteins, thus alleviating oxidative stress [104]. On the other hand, non-specific lipid transfer proteins (nsLTPs) contribute to lipid transfer, helping to maintain cell membrane stability and signal transduction, which counteracts the effects of salt stress [105]. In summary, the application of proteomics in studying beetroot root salt tolerance not only reveals the complex changes in proteins under salt stress but also highlights the intricate interactions between proteins in response to salt stress. These findings underscore potential targets for genetic engineering to enhance crop resilience and emphasize the importance of proteomic approaches in elucidating the mechanisms underlying beetroot root salt tolerance.

### 4.3. Post-Translational Modification Omics of Salt Tolerance in Sugar Beet

The proteomics of post-translational modifications (PTMs) are crucial for diversifying protein function, localization, stability, and interaction [106]. These modifications include phosphorylation, glycosylation, ubiquitination, and methylation, among others. In the sugar beet, PTM proteomics has emerged as a powerful tool to explore the mechanisms underlying salt tolerance. The N6-methyladenosine (m6A) methylation was used to reveal the effect of salt stress on sugar beet, with 80% of DEGs showing expression patterns negatively correlated with m6A modification. This regulation affects genes involved in energy metabolism, transport, signal transduction, transcription factor activity, and cell-wall organization [107]. The ubiquitination process serves as a critical mechanism for protein degradation and turnover, thereby maintaining cellular homeostasis under stress conditions. In sugar beet under salt stress, a large number of differentially ubiquitinated proteins were found, including those involved in cell transcription, translation, signal transduction, metabolic pathways, and the ubiquitin/26S proteasome pathway [76]. Additionally, reversible protein phosphorylation plays an important regulatory role in signaling, cell signal expansion, and cell cycle control. This reversible nature allows for rapid and precise adjustments to protein activity and function, enabling sugar beet to respond swiftly to environmental changes [72]. In summary, PTMs offer a profound perspective in the realm of salt tolerance in sugar beet. By unraveling the complex web of PTMs, scientists can better understand how sugar beet adapts to salt-stress conditions.

### 4.4. Metabolomics of Salt Tolerance in Sugar Beet

Metabolomics, the comprehensive study of small-molecule metabolites within living organisms, has significantly advanced the understanding of salt-tolerance mechanisms in sugar beet [74]. Using metabolomics, a TCA-related organic acid (OA) increase in sugar beet leaves under salt stress was found, along with the accumulation of proline, mannitol, and putrescine, which contribute to salt-stress adaptation, revealing how sugar beet modulates its metabolic pathways to enhance resilience [74]. A metabolomic analysis of sugar beet treated with 300 mM Na^+^ for 1 d and 7 d discovered that salt stress enhances sucrose metabolism and TCA cycle activity. After 1 d of stress, sucrose levels decreased while organic acids like l-malate and 2-oxoglutarate increased. After 7 d of stress, nitrogen-containing metabolites, including amino acids, betaine, melatonin, and (S)-2-aminobutyric acid, showed significant increases [49]. Additionally, other research has also revealed that salt stress reduces key metabolites involved in the Calvin cycle, glycolysis, and citric acid cycle [74]. Under salt stress, compounds such as arabinose, glycolic acid, inositol, malate, and mannitol accumulate both inside and outside chloroplasts [108]. This accumulation supports the chloroplast’s biochemical functions and enhances photorespiration metabolism, thus improving sugar beet’s adaptation to salt stress. These metabolites help maintain cellular osmotic balance and protect cellular structures from oxidative damage. Metabolomics reveals complex metabolite interactions in sugar beet, highlighting its importance in understanding and enhancing salt tolerance.

## 5. Outlook and the Future

Although progress has been made in understanding sugar beet salt tolerance, it is less frequently studied compared to food crops. Consequently, research on its salt-tolerance mechanisms remains limited, resulting in an incomplete understanding of its response to high salt environments. In contrast, Tamarix, a highly salt-tolerant plant, has been extensively studied. Tamarix is a known salt-secreting halophyte. As for sugar beet, the question remains open whether, under salinity stress, its adaptive mechanisms concentrate on salt exclusion, lean towards salt secretion, or perhaps incorporate both features. Tamarix can secrete various anionic ions and possesses different abilities to secrete these ions [109,110]. Some of the literature indicates that Tamarix salt-gland secretion is influenced by factors such as circadian rhythm [111], soil salt-ion content [112], temperature, humidity, and soil moisture. Similar experiments on sugar beet showed varying responses to different neutral salts, with NaCl having a more severe impact than Na_2_SO_4_ [49]. The underlying reasons remain unclear, but it is possible that the salt resistance mechanisms in sugar beet are influenced by similar factors as in Tamarix. Therefore, further in-depth research is needed to fully understand the salt-tolerance mechanisms in sugar beet.

Current omics studies have certain limitations. In transcriptomics, advancements such as single-cell transcriptomics and spatial transcriptomics provide more detailed insights. For instance, a study using single-cell transcriptomics constructed a transcriptional map of cotton root tips responding to salt stress at single-cell resolution, revealing cell heterogeneity, root type differences, and differentiation trajectories under salt stress. Similarly, in post-translational modifications, research has focused on acetylation and other modifications. One study found that *GmNFYA* regulates histone acetylation modifications, promoting the expression of downstream salt-tolerance genes and thereby enhancing soybean’s salt tolerance [113]. In metabolomics, broad-target metabolomics can provide comprehensive information on metabolic changes under stress conditions. Through targeted metabolomics, researchers have deeply analyzed metabolic changes in plants under salt stress and identified key metabolic pathways and genes involved in wheat’s response to salt stress [114]. All these areas require further in-depth research on sugar beet to fully understand its response to salt stress.

Multi-omics integrates genomics, transcriptomics, proteomics, post-translational modification omics, and metabolomics, fully revealing the complex biological processes in plants under salt stress. Significant progress has been made in multi-omics studies in other crops. Through genomics, transcriptomics, proteomics, and other multi-omics studies, research on rice salt tolerance has provided detailed mechanisms related to salt-stress tolerance, including salt sensing, ROS scavenging, cytoskeleton regulation, and protein synthesis [115]. Similarly, utilizing multi-omics approaches combining transcriptomics, proteomics, and metabolomics, researchers found that the phenylpropanoid pathway plays a crucial role in the relationship between growth, development, and secondary metabolites in roses under salt stress [116]. Additionally, by integrating analyses from microbiome, transcriptome, and metabolome studies, researchers identified key operational taxonomic units (OTUs), core genes, and important metabolites involved in Cynomorium songaricum’s response to salt stress [117]. These findings highlight the potential of multi-omics approaches in improving salt tolerance. Although sugar beet is more salt-tolerant than many crops, high salinity can still negatively impact its growth, yield, and quality. The application of omics technology provides new insights into the salt resistance mechanisms of sugar beet. To breed high-quality sugar beet salt-tolerance varieties, it is essential to identify salt resistance genes and understand the underlying mechanisms. Sugar beet has certain advantages in salt tolerance, such as its unique osmotic regulatory substance betaine. Revealing the salt-tolerance mechanisms of sugar beet can help us understand its adaptation strategies and provide references for improving salt tolerance in other crops. Advances in biotechnology are also noted, such as transgenic and gene-editing techniques. Transgenic and gene-editing technologies have shown tremendous potential in enhancing plant salt tolerance. These technologies allow scientists to precisely modify plant genomes by adding, deleting, or altering specific genes, thereby improving the plant’s ability to withstand environmental stress. Specifically, in staple crops like rice, extensive research has successfully used transgenic and gene editing technologies to enhance salt tolerance. For example, researchers have utilized CRISPR/Cas9 to target and edit the OsPIP1 gene in rice, significantly improving its growth under high-salt conditions [118]. Additionally, introducing salt-tolerance genes from salt-resistant plants, such as the SiMYB19 gene, into rice through transgenic techniques has significantly enhanced the growth and yield of transgenic rice under salt stress [119]. However, similar research on sugar beet has not been widely conducted. Currently, there is a lack of extensive studies applying transgenic and gene-editing technologies to improve the salt tolerance of sugar beet. Therefore, it is crucial to conduct further research in this area to fully utilize these advanced biotechnological tools to enhance the salt tolerance of sugar beet. This research is essential for the development of the sugar beet industry and for ensuring the crop’s productivity and quality in the face of increasing soil salinization and climate change challenges.

Despite a profound understanding of sugar beet responses to salinity stress at cellular and molecular levels under laboratory conditions, systematic studies on field experiments are still insufficient. Considering that laboratory tests often ignore the relationship between salt stress and sugar and yield, it is particularly critical to study how salt stress affects sugar beet sugar content, yield, and salt tolerance in field experiments. Moreover, the specific impacts of drip irrigation versus irrigation on sugar beet’s salt tolerance and disease incidence are still a largely unexplored territory. How these two irrigation methods specifically affect sugar beet’s growth, salt tolerance, and disease incidence is especially critical, given the nationwide threats posed by climate change, including potential expansions of saline soils and increased occurrences of extreme weather events, which present unprecedented challenges to sugar beet cultivation. Saline stress not only hinders sugar beet growth directly but may also indirectly elevate its disease incidence by altering the soil microenvironment and influencing pathogen activity, thereby imposing a dual threat to sugar beet production. Considering the economic value of sugar beet as a high sugar crop and its potential in saline land, exploring the effects of drip irrigation and irrigation on sugar content, yield, and disease resistance will help to optimize irrigation strategies and improve the adaptability and economic benefits of sugar beet.

As a specialized crop, sugar beet’s salt-tolerance research lags behind due to a lower academic focus and the scarcity of pertinent literature and resources, compounded by its intricate physiological mechanisms and interdisciplinary research complexities. Compared to mainstream crops, beet’s salt resistance studies appear more difficult and slow, particularly in terms of comprehensive experimentation and adaptive strategy exploration. In summary, with the severe date of soil salinity worldwide, the study of salt-tolerant crops is crucial. As an important salt-tolerance economic crop and sugar crop, sugar beet is important for its salt-tolerance gene mining and the analysis of its salt-tolerance mechanisms.

## 6. Conclusions

This article systematically investigates the adaptive mechanisms of sugar beet under salinity stress, elucidating its multidimensional responses at the physiological, cellular, and molecular levels. The findings reveal that sugar beet displays robust salt tolerance through mechanisms including ion regulation, enhanced antioxidant capacity, and metabolic acclimatization. Key genes and pathways identified via omics technologies pave the way for precision breeding and biotechnological applications in the future. To gain a more comprehensive understanding of sugar beet’s salt-tolerance mechanisms, we summarize major omics studies conducted using various technological platforms (Table 1).

Spanning transcriptomics, proteomics, and metabolomics, the studies not only furnish critical data underpinning a deeper insight into sugar beet’s physiological responses and adaptive strategies under saline conditions, but also offer novel strategies for enhancing sugar beet productivity and quality. These findings provide essential references for improving salt tolerance in other crops, thereby broadening the agricultural applications and resilience of various plant species against environmental stresses.

## Figures and Tables

**Table 1:** Major omic studies of salt-stress tolerance in sugar beet using different technological platforms.

Omics	Tissue	Reference	Highlights	Common Description
transcriptomics	Leaves	[52]	*aldh2b7*, *thic* and *delta-oat*	Under salt stress, multiple salt-tolerance-related genes in sugar beet are upregulated, particularly those associated with ion transport, antioxidant defense, and osmotic regulation, indicating its adaptive mechanisms in response to salt stress.
	Leaves and roots	[98]	*BvALKBH10B*	
	Leaves, roots	[120]	*BvM14-SAMDC*	
proteomics	Leaves	[53]	L-ascorbate oxidase	Salt stress significantly increases the expression of antioxidant enzymes and other key proteins, demonstrating that sugar beet responds to salt stress by enhancing its antioxidant capacity and regulating protein functions.
	Leaves and roots	[121]	BvARF	
	Leaves	[100]	PIP	
	Leaves and roots	[97]	psbQ-like protein 1, Plastocyanin and NAD(P)H quinone oxidoreductase subunit U	
	Leaves and roots	[103]	6-phosphofructokinase 5 (PFK5), malate dehydrogenase (MDH)	
	Leaves	[105]	non-specific lipid transfer proteins (nsLTPs)	
	Leaves	[72]	14-3-3	
	Roots	[76]	RUB1	
Metabolomics	Roots	[80]	The metabolism of carbon and nitrogen	Under salt-stress conditions, sugar beet accumulates various organic acids, proline, and other nitrogen-containing metabolites, enhancing its salt tolerance and demonstrating its adaptability in metabolic regulation.
	Leaves and roots	[49]	nitrogen-containing metabolites, including amino acids, betaine, melatonin, and (S)-2-aminobutyric acid	
	Leaves	[108]	arabinose, glycolic acid, inositol, malate, and mannitol	
	Leaves and roots	[96]	starch and sucrose metabolism, alpha-linolenic acid metabolism, phenylpropanoid biosynthesis	

## Data Availability

Data sharing is not applicable as no new data were generated or analyzed during this study.
